# Rare Adult Ileo–Ileo–Colic Intussusception Caused by an Ileal Lipoma with Spontaneous Ileal Rupture and Rectal Hemorrhage: A Case Report with a Hypothesis-Generating Discussion of Mesenteric Anatomical Variability

**DOI:** 10.3390/reports9030293

**Published:** 2026-09-01

**Authors:** Salvatore Pezzino, Mariacarla Castorina, Stefano Puleo, Sergio Castorina

**Affiliations:** 1 Department of Medicine and Surgery, University of Enna “Kore”, 94100 Enna, Italy; salvatore.pezzino@unikore.it; 2 Mediterranean Foundation “G.B. Morgagni”, 95125 Catania, Italy; 3 Department of Medical, Surgical Sciences and Advanced Technologies “G.F. Ingrassia”, University of Catania, 95123 Catania, Italy

**Keywords:** adult, ileum–ileum–colon, intussusception, lipoma, mesenteric anatomy, case report

## Abstract

**Background and Clinical Significance:** Adult intussusception is rare, accounting for approximately 5% of all intussusceptions and 1–5% of adult bowel obstructions, whereas the ileo–ileo–colic subtype is exceptionally uncommon. We report a rare case of ileo–ileo–colic intussusception caused by an ileal lipoma and complicated by spontaneous ileal rupture and rectal hemorrhage, emphasizing the diagnostic challenge and proposing the hypothesis that mesenteric anatomical variability may have contributed to its pathophysiology. **Case Presentation:** A 61-year-old man presented with progressive abdominal distension, right lower quadrant pain and mass, and signs of bowel obstruction. Contrast-enhanced abdominal CT showed an ileo–ileo–colic intussusception extending into the ascending colon, with a typical target sign and marked ischemic changes involving the ileal loops. Emergency laparotomy confirmed a complex ileo–ileo–colic intussusception extending into the right colon, with an ileal lipoma acting as the lead point. The intussuscepted bowel had undergone spontaneous rupture with active bleeding, explaining the rectal hemorrhage. The patient underwent right hemicolectomy with ileocecal resection and side-to-side ileocolic anastomosis. Histopathological examination confirmed a submucosal ileal lipoma associated with transmucosal ischemic necrosis. Recovery was uneventful, and no recurrence or late complications were observed at 6- and 12-month follow-up. **Conclusions:** This case highlights an exceptionally rare and severe presentation of adult intussusception. Early CT diagnosis and prompt surgical resection are essential when ischemia, necrosis, or failed reduction are present. Mesenteric anatomical variability is proposed as a plausible contributing factor to the extensive telescoping and vascular compromise observed in this patient, a hypothesis that warrants further anatomical investigation.

## 1. Introduction and Clinical Significance

Intussusception is defined as the invagination or telescoping of one segment of the gastrointestinal tract and its mesentery (intussusceptum) into the lumen of an adjacent distal segment (intussuscipiens). While this condition represents the most common abdominal surgical emergency in children, it is extremely rare in adults, accounting for approximately 5% of all cases and 1–5% of intestinal obstructions in the adult population, with a child-to-adult ratio of nearly 20:1 [1,2]. Unlike in children, where idiopathic causes dominate in up to 80–95% of cases, approximately 90% of adult intussusceptions are secondary to identifiable pathological lead points, with two-thirds resulting from benign or malignant neoplasia [1,2].

Intestinal intussusception is classified anatomically into ileocecal, ileocolic, ileo–ileo–colic, ileo–ileal, and colocolic types. Among operated adult patients, a recent systematic review of 37 studies (2330 patients) reported that 65% of cases were enteric (small bowel), 21% ileocolic, and 20% colocolic [3]. The ileo–ileo–colic variant reported here represents fewer than 1% of all adult intussusceptions and is characterized by the telescoping of an ileal segment with its mesentery first into the distal ileum and subsequently across the ileocecal valve into the colon, involving complex mesenteric folding and vascular entrapment [4,5].

The probability of underlying malignancy varies markedly between subtypes: colocolic intussusceptions harbor malignancy in up to 68% of cases, ileocolic forms in approximately 46%, and small-bowel intussusceptions in only 9% [3,6]. This distinction has direct implications for surgical decision-making, as colonic variants should be approached with oncological intent and formal resection without prior reduction [6].

Intestinal lipomas are rare benign mesenchymal tumors representing 2–5% of all gastrointestinal tumors with 60–70% located in the colon and ileum–colon region [7]. Approximately 75% of lipomas larger than 2 cm become symptomatic, causing pain, gastrointestinal bleeding, intussusception, or obstruction [7,8,9]. As a cause of adult intussusception, lipomas account for approximately 6% of cases but remain among the most frequent benign lead points in systematic series [3,6,8], acting as a pathological “battering ram” that provides the mass and mobility necessary to initiate and sustain telescoping [10,11].

**Figure 1:**
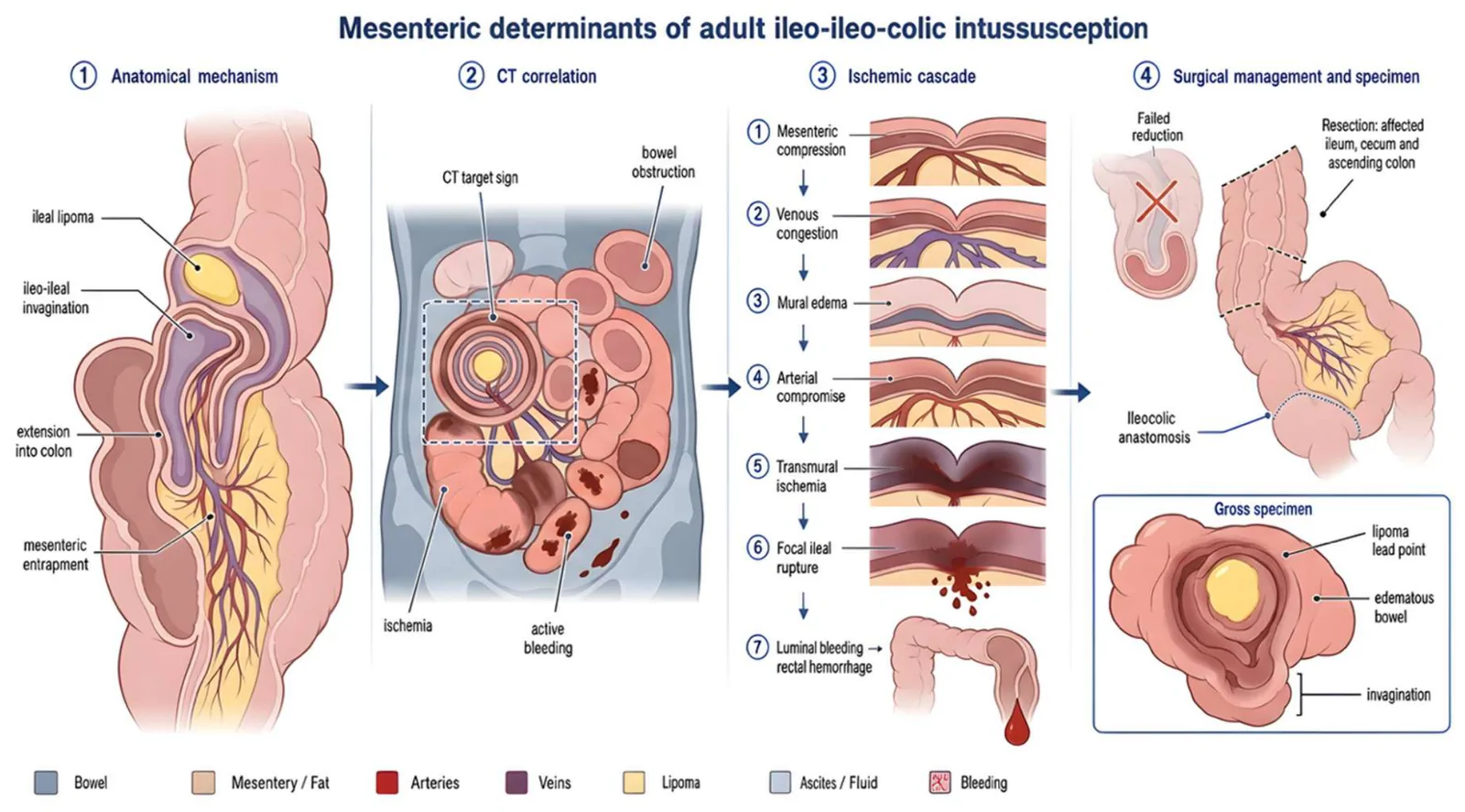
Schematic representation of adult ileo-ileo-colic intussusception secondary to an ileal lipoma. The left panel depicts the lipoma acting as the pathological lead point, with telescoping of the ileal segment and its mesentery into the distal bowel, forming the characteristic concentric layers of the intussusception complex. The central panel reproduces the typical axial CT appearance, showing the “target” or “bull’s-eye” sign with a central lipomatous lesion and entrapped mesenteric vessels. The vertical sequence illustrates the pathophysiological progression from early mesenteric compression and venous congestion to mural edema, vascular compromise, transmural ischemia, necrosis, and eventual rupture with intraluminal hemorrhage. The upper-right panel highlights the surgical strategy, emphasizing avoidance of forceful reduction and performance of formal resection, whereas the lower-right panel shows the gross specimen with the concentric intussusception pattern and the lipoma visible at the center of the lumen.

As the bowel telescopes, the mesentery and its neurovascular structures become progressively entrapped, leading to vascular compromise and ischemic injury [12,13,14]. Individual variation in mesenteric length, mobility, and attachment pattern has been proposed in anatomical and pediatric surgical literature as a factor that may influence the extent of telescoping once a lead point is present [12,13,14], raising the question of whether such variability may also contribute to the severity of complex adult presentations (Figure 1).

We present a rare case of adult ileo–ileo–colic intussusception caused by an intestinal lipoma acting as a lead point. This report focuses on the anatomical analysis of mesenteric involvement and its role in the complex pathophysiology of this exceptionally rare variant, contributing to the limited existing literature on ileo–ileo–colic intussusception and exploring the hypothesis that mesenteric anatomical variability may have contributed to the extensive telescoping and vascular compromise observed in this patient.

## 2. Case Presentation

### 2.1. Patient Information and Physical Examination

A 61-year-old male (born 1964) was referred to our department with a several-day history of absent feces and flatus, progressive abdominal distension, a sensation of abdominal tension, and moderate spontaneous pain in the right iliac fossa. Digital rectal examination was negative. Subsequently, the patient experienced a single episode of hematochezia of approximately 300 mL, without hemodynamic instability. His past medical and surgical history was otherwise unremarkable; he reported no known drug allergies or intolerances. Relevant medical history included arterial hypertension treated with bisoprolol fumarate. Differential diagnoses at presentation included cecal carcinoma and perityphlitic abscess. On admission, he was in fair general condition, alert and oriented, with a temperature of 37.5 °C, blood pressure of 155/90 mmHg, arterial PaO2 of 191 mmHg, heart rate of 96 beats/min with regular cardiac activity, and respiratory rate of 17 breaths/min. He remained hemodynamically stable. Abdominal examination revealed a globose abdomen without signs of peritonitis on initial assessment; palpation demonstrated a poorly defined, firm, hard–elastic, and tender mass in the right iliac fossa.

### 2.2. Diagnostic Investigations

Laboratory testing indicated hemoglobin of 15.7 g/dL and RBC count of 4,990,000/mm^3^, with intact erythrocytic parameters and no anemia. The WBC count was considerably high at 20,500/mm^3^ with 88.4% neutrophils, consistent with an acute inflammatory disease. C-reactive protein was significantly elevated at 105 mg/L (normal range < 5 mg/L), further supporting the systemic inflammatory response. Platelet count was within normal range at 245,000/mm^3^. The metabolic indices showed a blood glucose level of 107 mg/dL and a creatinine level of 1.35 mg/dL with a BUN of 78.5 mg/dL, indicating a probable prerenal involvement due to dehydration rather than overt renal failure. Liver function tests were unremarkable (ALT 8.36 U/L; AST 22 U/L). Coagulation parameters (PT 11.4 s, 97%; INR 1.02) were within normal limits. Fibrinogen was high at 524 mg/dL, consistent with an acute phase reaction. Electrolytes, GGT, ALP and lactate were within non-pathological ranges, arguing against significant metabolic derangement or extensive tissue hypoperfusion at the time of presentation.

### 2.3. CT Imaging Findings

**Figure 2:**
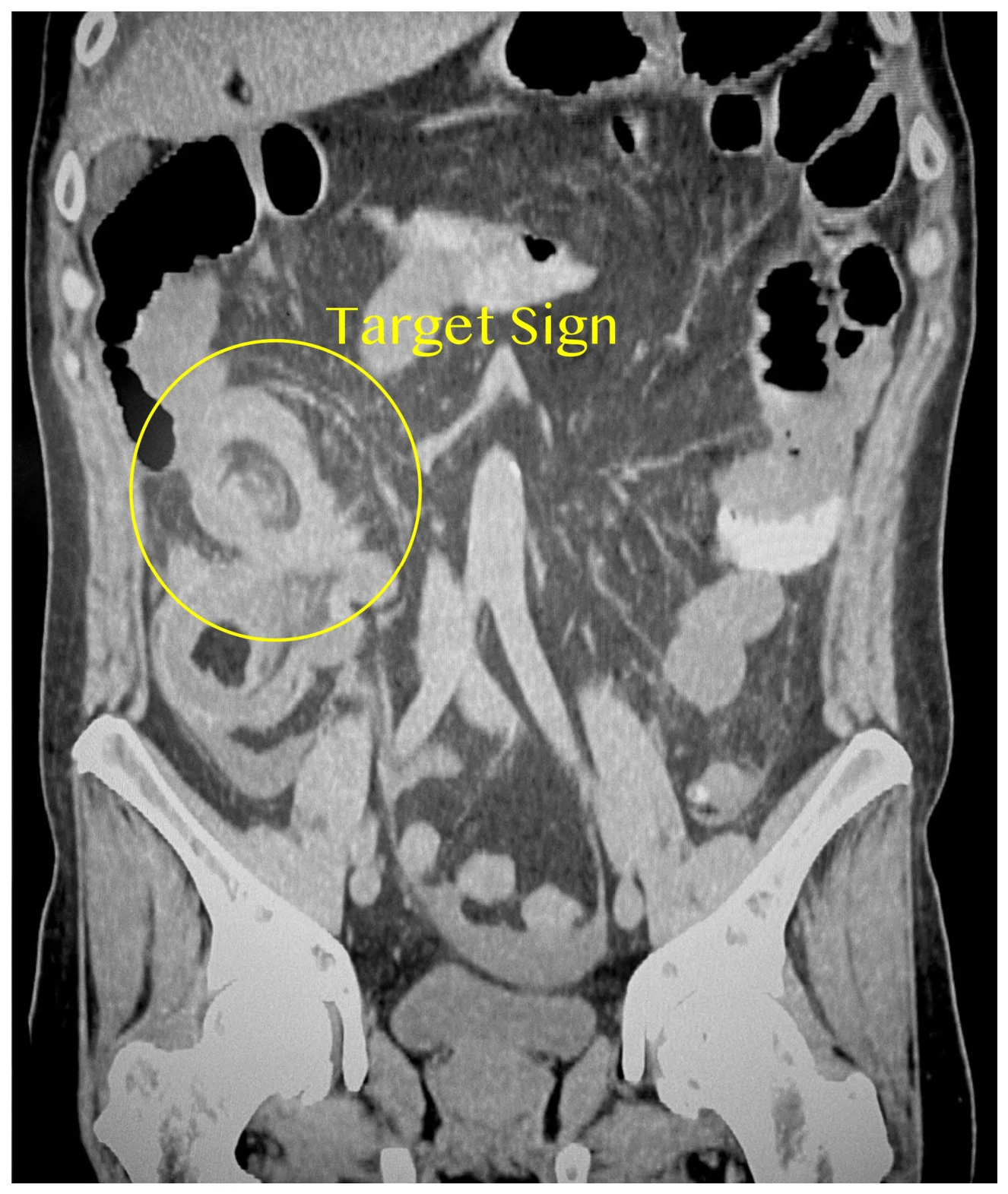
Coronal contrast-enhanced CT image of the abdomen showing ileo–ileo–colic intussusception occupying the ascending colon. The circle identifies the characteristic target configuration, formed by concentric bowel-wall layers with central mesenteric fat and entrapped vessels. The markedly thickened ileal loops demonstrate objective signs of severe ischemic compromise (mural thickening, hypoattenuation, mesenteric fat stranding), without direct demonstration of active contrast extravasation due to the absence of a dedicated arterial-phase acquisition.

Contrast-enhanced abdominal CT revealed ileo–ileo–colic intussusception occupying the ascending colon with associated bowel obstruction. The characteristic ‘target sign’ (also described as ‘bull’s eye sign’ or concentric circle sign) was demonstrated, with central mesenteric fat and vessels drawn into the intussuscepted segment. The ileum showed severe ischemic compromise, with hyperdense intraluminal/intramural content suggestive of a hemorrhagic component; however, the CT protocol did not include a dedicated arterial phase, precluding definitive demonstration of active contrast extravasation. CT also demonstrated a small amount of ascitic fluid, ascending ileocecal intussusception, a distended colonic frame, and multiple ileal hydro-aerial levels in the mid-abdomen (Figure 2). The extent of mesenteric involvement and degree of telescoping observed on imaging suggested anatomical predisposition with increased mesenteric mobility facilitating the complex invagination process. CT is regarded as the gold standard for diagnosing intussusception in adults, with a preoperative diagnostic accuracy of 82% confirmed by a 2025 systematic review; sensitivity exceeds 85% with modern multislice scanners [6,15].

### 2.4. Therapeutic Intervention, Operative Findings, and Outcomes

The patient was diagnosed with ileo–ileo–colic intussusception with intestinal obstruction and underwent emergency laparotomy under general anesthesia.

**Intraoperative findings:** At laparotomy, an ileo–ileo–colic intussusception was confirmed. The intussusceptum measured approximately 12 cm and was contained within an approximately 20 cm intussuscipiens segment. The ileal lipoma acting as the lead point was located approximately 10 cm proximal to the ileocecal valve. The colonic component of the intussusception extended into the ascending colon, reaching the hepatic flexure. A gentle and brief attempt at reduction was made. Because colonoscopy had documented a mucosal lesion before surgery, operative manipulation was excluded as the cause of the bowel wall defect. The mesenteric anatomy showed marked folding and vascular compromise related to the telescoping mechanism.

**Surgical procedure:** Initial attempts at reduction in the intussusception were unsuccessful. Surgical management included resection of the ileocecal region and right hemicolectomy, followed by manual side-to-side ileocolic anastomosis using 4-0 PDS sutures. The decision for formal resection rather than simple reduction was guided by the widespread mesenteric involvement, vascular compromise, and intraoperative evidence of intestinal necrosis. Perioperative antibiotic therapy with cefazolin and metronidazole was administered and continued until postoperative day 3, in accordance with institutional management protocols for contaminated surgery.

**Figure 3:**
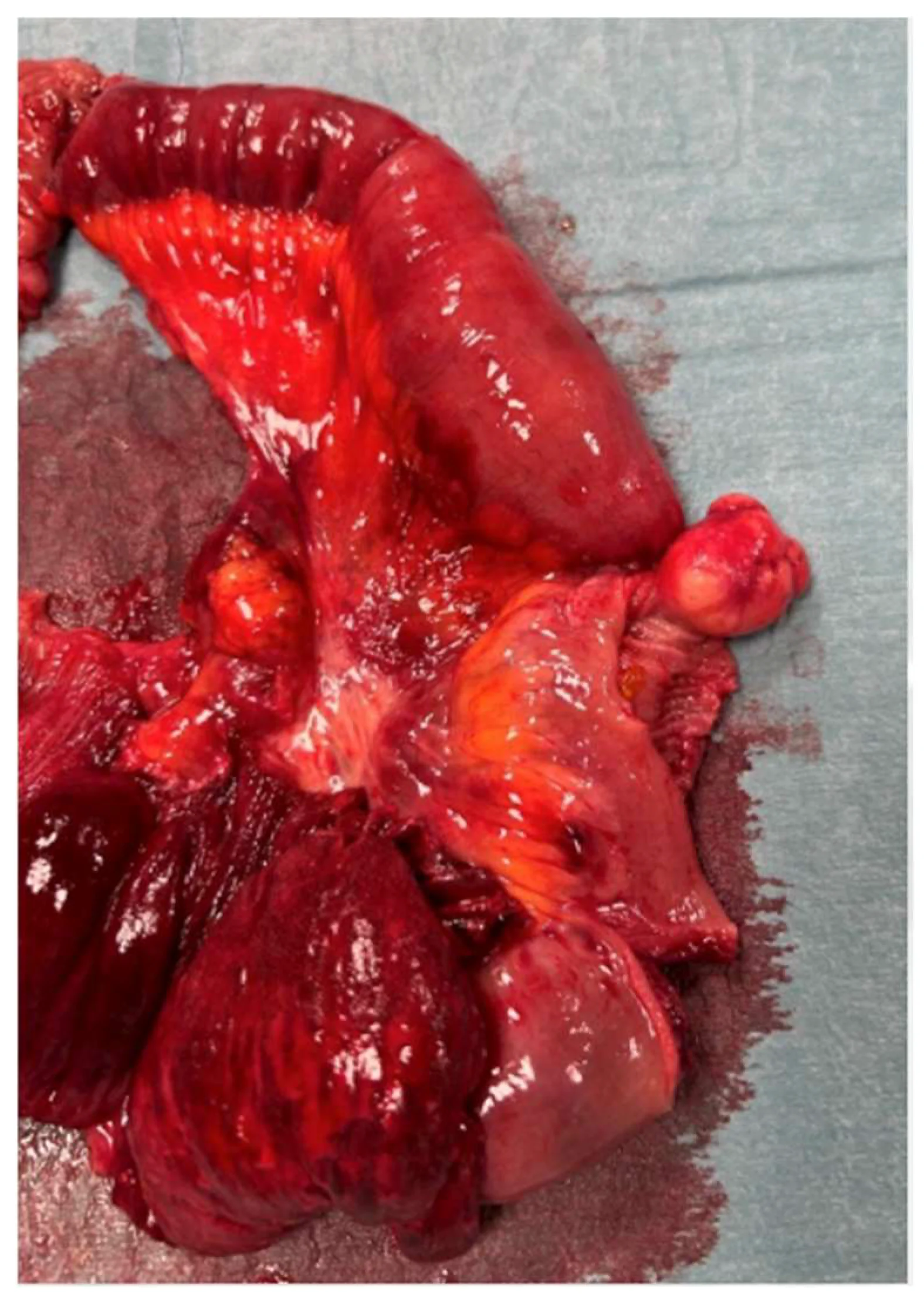
Gross specimen of the ileo–ileo–colic intussusception. The resected segment shows a markedly congested and edematous ileal loop invaginated into the cecocolic segment, with extensive hemorrhagic serosal discoloration consistent with severe ischemia and impending transmural necrosis. A well-circumscribed, polypoid, yellow adipose mass arising from the ileal wall represents the submucosal lipoma acting as the pathological lead point.

**Macroscopic examination:** Macroscopic examination of the resected specimen revealed a full-thickness longitudinal laceration along the antimesenteric border of the cecum, with hemorrhagic wound margins. The lesion was locally confined, with no evidence of free enteric spillage, hemoperitoneum, or pneumoperitoneum. The gross specimen revealed intestinal wall congestion and edema throughout the affected segments. Within the small bowel loop inside the cecum, extensive necrotic areas were identified with partial wall rupture, explaining the preoperative bleeding. The mesenteric vessels showed severe compression; macroscopic thrombosis was suspected, although it was not confirmed histologically (Figure 3). Notably, the invaginated ileum had undergone spontaneous rupture with bleeding, resulting in frank rectal hemorrhage.

**Histopathological examination:** The surgical resection specimen consisted of 18 cm of ileum, cecum, and 26 cm of colon. Between the ileum and cecum, a 3-cm polypoid lesion with adipose appearance was identified. An area of mucosal intussusception of the right colon with extension of less than 10 cm was also observed. Final diagnosis: 3-cm submucosal lipoma with transmucosal ischemic necrosis of the ileal mucosa. The cecal appendix and five sampled lymph nodes were unremarkable.

**Postoperative course and follow-up:** The postoperative course was uneventful. The patient was discharged on postoperative day 7. Clinical evaluation and abdominal imaging at 6 and 12 months showed no evidence of late complications; at the last follow-up visit, he was in good general condition with preserved quality of life.

The chronological sequence of clinical presentation, diagnostic work-up, treatment, and follow-up is summarized in Table 1.

## 3. Discussion

Adult intussusception is rare, accounting for approximately 5% of all intussusception cases and 1–5% of adult bowel obstructions [2,6,12]. Its presentation is typically insidious and nonspecific, in contrast with the classic pediatric triad of colicky pain, ‘currant-jelly’ stool, and a palpable mass, which is infrequently observed in adults. A large retrospective analysis of 4432 patients from the National Inpatient Sample demonstrated that adult mortality is only 0.1%, but each additional day of surgical delay raises mortality odds by 26.7% (OR 1.267; 95% CI 1.053–1.525; *p* = 0.012), underscoring the urgency of timely intervention [16].

Malignancy risk differs sharply across anatomical subtypes: colocolic intussusceptions harbor malignancy in up to 68% of cases, ileocolic forms in approximately 46%, and small-bowel intussusceptions in only 9% [17].

The ileo–ileo–colic variant observed here, representing less than 1% of all adult intussusceptions, combines this diagnostic uncertainty with dual-level mesenteric vascular compromise, which is the key feature distinguishing this case from more common ileocolic or colocolic presentations.

In this case, an intestinal lipoma served as the pathological lead point, accounting for approximately 6% of adult intussusception cases yet remaining one of the most frequent benign lead points in systematic series [18,19]. Its antimesenteric implantation and soft, pliable consistency allowed it to act as a mobile ‘battering ram’, sufficient to drive invagination through the ileocecal valve—a landmark that typically resists telescoping—and into the ascending colon. Recent case series have similarly documented the capacity of ileal lipomas to cause simultaneous hemorrhage and obstruction [20,21,22]; in the present case, spontaneous transmural rupture of the invaginated ileum produced frank rectal bleeding, a rare but recognized complication.

CT characterizes lipomas with reasonable confidence owing to their fat attenuation, informing the likelihood of benign rather than malignant etiology, and remains the most widely accepted diagnostic tool for adult intussusception [15,23], with a 2025 systematic review confirming a preoperative diagnostic accuracy of 82%, compared with 54% for ultrasound and 74% for colonoscopy in colonic cases [6]. Modern multislice CT series report accuracy ranging from 78% to 100%, with characteristic findings including the “target sign,” “doughnut sign,” or “bull’s-eye sign” reflecting the concentric arrangement of bowel walls, and the “sausage-shaped” mass on longitudinal sections [24,25]. Despite this diagnostic superiority, a radiological lead point was described in only 31% of patients in the largest systematic review, highlighting the persistent preoperative diagnostic challenge [6,25]. Ultrasound, while noninvasive and radiation-free, demonstrates substantially lower sensitivity in adults compared with near-100% sensitivity in children, improving to approximately 91% when a palpable abdominal mass is present [26,27,28].

Understanding mesenteric anatomy is fundamental to the pathophysiology of ileo–ileo–colic intussusception. As the bowel telescopes, the mesentery undergoes sequential structural compromise—primary folding during ileum-to-ileum invagination, secondary compression at the ileocecal valve, and tertiary vascular entrapment through dual-level angulation [13,14]. Low-pressure venous channels fail first, causing congestion and mural edema; arterial compromise follows, and lymphatic obstruction amplifies wall edema and local inflammation. If ischemia is not promptly relieved, the cascade culminates in transmural necrosis, bacterial translocation, and potentially perforation with septic shock [13,14]. CT findings of hypodense layers in the returning wall, perilesional fluid, and intramural gas can predict the degree of vascular compromise and guide urgency of operative intervention.

A critical observation in this case concerns individual mesenteric variability as a predisposing factor. The mere presence of a lipoma is insufficient to account for intussusception, given that these benign tumors are relatively common yet rarely cause telescoping. Patients with longer, more mobile mesenteric attachments or a broader mesenteric root may show increased susceptibility when a pathological lead point is present; the configuration of the mesenteric root dictates the extent and direction of telescoping, explaining the paradox of extensive invagination here while similar-sized lipomas remain clinically silent in others.

Current evidence-based guidance from a 2025 systematic review recommends en bloc oncological resection without prior reduction for colocolic or ileocolic intussusceptions with high malignancy suspicion, to prevent intraluminal seeding or venous tumor dissemination [6,29]. For small-bowel intussusceptions with a definitively benign etiology, intraoperative reduction before resection may be considered to preserve bowel length; if reduction fails, immediate en bloc resection is indicated. In the present case, right hemicolectomy was performed due to severe mesenteric involvement, vascular compromise, and evidence of intestinal necrosis [6,29]. The complex mesenteric entrapment characteristic of this variant precludes simple reduction, as the attempt risks mesenteric avulsion or incomplete decompression of compromised vascular structures. Open laparotomy, as performed here, remains the approach of choice in patients with bowel ischemia or complex mesenteric involvement.

Adult surgical management contrasts sharply with pediatric ileocolic intussusception, where image-guided (ultrasound- or fluoroscopy-guided) hydrostatic or pneumatic reduction is the established first-line treatment, achieving success rates exceeding 80–90% without surgery, since idiopathic lymphoid hyperplasia rather than a fixed pathological lead point is the usual underlying cause. Recent evidence indicates that ultrasound-guided hydrostatic reduction can achieve comparable success rates whether performed by pediatric radiologists or by appropriately trained non-pediatric radiologists, sonographers, or residents, underscoring that prompt diagnosis—rather than operator subspecialization alone—is the critical determinant of successful nonoperative management in children, and that this diagnostic step can be reliably performed even by less experienced sonographers once adequately trained [30]. This paradigm stands in direct contrast to the adult presentation described here: the presence of a fixed anatomical lead point (the lipoma), the associated risk of malignancy, and the mesenteric vascular compromise observed intraoperatively make image-guided nonoperative reduction inappropriate in this setting, and surgical resection remains the standard of care in adults regardless of how promptly the diagnosis is established or how experienced the operator performing the initial ultrasound may be.

Ileo–ileo–colic intussusception caused by an ileal lipoma is exceedingly rare; only a handful of analogous cases have been reported [19,31,32,33]. The present case is further distinguished by spontaneous rupture of the invaginated ileum with frank rectal hemorrhage, a complication documented in very few cases of lipoma-induced intussusception [11,34,35]. Chiu et al. (2022) described a combined ileo–ileal and ileocolic intussusception secondary to an inflammatory fibroid polyp in an adult, illustrating the diagnostic and anatomical complexity inherent to dual-level intussusception [36]. Badshah et al. (2025) [37] reported ileocecal intussusception secondary to a giant ileal lipoma associated with chronic constipation, confirming surgical resection as the treatment of choice when bowel compromise is present. Recent Italian experience further corroborates the value of a multidisciplinary approach in managing hemorrhagic lipoma-induced intussusception [35].

To place these observations in sharper comparative perspective, three differences from the existing literature deserve emphasis. Regarding rare causes, the ileo–ileo–colic subtype itself remains exceptionally uncommon, representing fewer than 1% of adult intussusceptions, whereas the great majority of previously reported lipoma-associated cases—including those of Waack et al. [10], Ramzan and Jain [11], and Balamoun and Doughan [31]—involve the simpler, single-level ileocolic or colocolic patterns; the dual-level mesenteric involvement described here is therefore anatomically distinct from most of the comparator literature. Regarding clinical presentation, spontaneous transmural rupture with overt rectal hemorrhage, as observed in this patient, stands in contrast to the more indolent, obstructive, or chronic intermittent symptomatology reported by Chiu et al. [36] and Badshah et al. [37], and is more closely—though not identically—approximated by the occult or intermittent bleeding described by Valeriani et al. [35]; free intraluminal rupture of this severity remains a distinctly uncommon finding even within this subset of hemorrhagic presentations. Regarding treatment, while several prior cases were managed with elective or semi-elective segmental resection once a benign lead point was confirmed [11,31], the combination of dual-level vascular compromise and active hemorrhage in the present case precluded any trial of definitive nonoperative or delayed management and required emergency en bloc resection, underscoring how the severity of the anatomical and vascular derangement, rather than the benign histology of the lead point alone, ultimately dictated the surgical urgency.

This report has several inherent limitations. As a single case report, generalizability is limited by definition. No preoperative imaging or quantitative data on mesenteric variability were available to formally characterize the anatomical predisposition, which remains a hypothesis supported by intraoperative and imaging observations. There was limited follow-up, although a 1-year follow-up is adequate for a benign condition. Furthermore, the absence of prospective multicenter data on the ileo–ileo–colic subtype means that the surgical algorithm proposed here is based on institutional practice and expert consensus rather than high-level evidence. No histological slides of sufficient quality for publication were available from the archived pathology material, as the specimen was processed according to routine diagnostic protocols without dedicated research-grade imaging; this precluded the inclusion of an H&E figure, which we acknowledge as a limitation of the present report. In addition, the CT protocol did not include a phase optimized to demonstrate active contrast extravasation, limiting direct radiological confirmation of active bleeding. Mesenteric vascular thrombosis was suspected macroscopically but was not confirmed by histological examination. These issues represent further limitations of the present report.

## 4. Conclusions

Ileo–ileo–colic intussusception caused by an intestinal lipoma in adults is an exceedingly rare condition that presents significant diagnostic and therapeutic challenges. This case demonstrates the critical importance of mesenteric anatomy in the pathophysiology of adult intussusception: the lipoma’s ‘battering ram’ function creates complex mesenteric folding and vascular compromise across multiple intestinal segments.

The extent of telescoping observed in this case leads us to propose that individual variation in mesenteric anatomy may plausibly contribute to the severity of such presentations, a hypothesis that merits dedicated anatomical investigation.

CT, with a preoperative diagnostic accuracy of 82% in the most recent systematic series, remains the gold standard for preoperative diagnosis and assessment of ischemic complications. Timely surgical intervention with formal resection is essential when extensive mesenteric involvement and vascular compromise are present, while efforts should focus on preserving as much functional bowel as possible. Recognition of these anatomical principles is essential for optimal surgical planning, and this case offers a hypothesis-generating contribution toward understanding why comparable lead points produce such divergent clinical presentations.

## Figures and Tables

**Table 1:** CARE-compliant timeline of clinical presentation, treatment, and follow-up.

Timepoint	Clinical Event
**Several days before admission**	Progressive partial obstipation, abdominal distension, abdominal tension, and moderate spontaneous right iliac fossa pain
**Day 0**	Hospital admission. Digital rectal examination was unremarkable. The patient subsequently experienced a single episode of hematochezia of approximately 300 mL, without hemodynamic instability
**Day 0**	Laboratory investigations showed leukocytosis and elevated C-reactive protein, without anemia; contrast-enhanced abdominal CT demonstrated ileo–ileo–colic intussusception involving the ascending colon, with bowel obstruction and imaging findings suggestive of ischemic injury
**Day 1**	Emergency laparotomy confirmed complex ileo–ileo–colic intussusception caused by an ileal lipoma, associated with ischemic bowel injury and a full-thickness bowel wall defect
**Day 1**	Right hemicolectomy with ileocecal resection and manual side-to-side ileocolic anastomosis
**Postoperative days 1–3**	Cefazolin and metronidazole administered as postoperative antibiotic therapy
**Day 7**	Uneventful postoperative recovery and hospital discharge
**Month 6**	Clinical and abdominal imaging follow-up: no recurrence or late complications
**Month 12**	Clinical and abdominal imaging follow-up: no recurrence or late complications; patient in good general condition

## Data Availability

The authors confirm that the data supporting the findings of this study are available within the article.

## Abbreviations

The following abbreviations are used in this manuscript:

ALPI	Alkaline phosphatase
ALT	Alanine aminotransferase
AST	Aspartate aminotransferase
BUN	Blood urea nitrogen
CRP	C-reactive protein
CT	Computed tomography
INR	International normalized ratio
PDS	Polydioxanone suture
PT	Prothrombin time
RBC	Red blood cell count
WBC	White blood cell count
